# The Cervical Cancer Paradox in Eastern Europe: How Knowledge and Institutional Trust Shape HPV Vaccination Attitudes in Romania

**DOI:** 10.3390/medsci14040431

**Published:** 2026-07-25

**Authors:** Ionel-Daniel Nati, Carmen Mihaela Mihu, Dan Mihu, Razvan Ciortea, Doru Diculescu, Mihaela Oancea, Carmen Bucuri, Maria Patricia Roman, Cristina Mihaela Ormindean, Viorela Suciu, Dumitru Rares Ciocoi-Pop, Andrei Mihai Malutan

**Affiliations:** 1 2nd Department of Obstetric and Ginecology, “Iuliu Hatieganu” University of Medicine and Pharmacy, 400610 Cluj-Napoca, Romania; ionel.nati@umfcluj.ro (I.-D.N.);; 2 Department of Morphofunctional Sciences, Iuliu Hațieganu University of Medicine and Pharmacy, 400012 Cluj-Napoca, Romania; 3 Physical Therapy and Theoretical Subjects Department, Babeș-Bolyai University of Cluj Napoca, 400347 Cluj-Napoca, Romania

**Keywords:** cervical cancer, HPV vaccination, health literacy, institutional trust, misinformation, vaccine hesitancy

## Abstract

Background: Although cervical cancer is a highly preventable malignancy, Romania continues to report critical mortality rates. This paradox is largely attributable to the suboptimal implementation of clinical guidelines and a passive, “opt-in” administrative framework. This cross-sectional study investigates the cognitive and behavioural determinants—specifically, human papillomavirus (HPV) knowledge, institutional trust in healthcare systems, and digital misinformation exposure—that modulate female attitudes toward HPV immunisation. Methods: An observational cross-sectional study was conducted between December 2025 and March 2026 across four obstetrics and gynaecology clinics. Data were collected via digital questionnaires administered to adult female patients aged 18 to 65 years. The assessment tool evaluated four primary domains: HPV knowledge, perceived vaccine safety, immunisation intention, and the degree of exposure to social media misinformation. Results: The cohort demonstrated a substantially low vaccination rate (28.6%). Statistical analysis elucidated significant positive correlations between HPV knowledge, trust in health authorities, and vaccination intention. Furthermore, reliance on official information sources strongly correlated with perceived vaccine safety. Conversely, exposure to negative online content exhibited a weak negative association with knowledge levels, yet it did not directly influence vaccination intention. Conclusions: Health literacy and institutional trust emerge as fundamental pillars for HPV vaccine acceptance. To overcome patient vaccine hesitancy, the national healthcare system must initiate a strategic paradigm shift, prioritising the neutralization of digital misinformation and transitioning from a passive model to a proactive, “opt-out” infrastructure.

## 1. Introduction

Cervical cancer is a malignancy characterised by its near-total preventability through prophylactic vaccination and organised screening, even so, it remains a persistent public health crisis in settings where clinical guidelines are suboptimally implemented, most notably in several Central and Eastern European countries—including Romania, which records the highest cervical cancer incidence and mortality in the European Union [1].

The epidemiological burden of the disease is linked to the prevalence of the Human Papillomavirus (HPV), a high-risk pathogen whose oncogenic potential is not limited to the cervix [2]. Indeed, HPV is a multisystemic driver of carcinogenesis, contributing to approximately 5% of the global neoplastic burden across a diverse spectrum of anatomical sites [2,3,4,5]. Beyond cervical cancer, HPV has been causally linked to a substantial proportion of vulvar, vaginal, anal, penile, and oropharyngeal (head and neck) cancers, underscoring its role as a multisystem oncogenic driver affecting both sexes. Penile cancer, although less frequently discussed in public health messaging—partly owing to its lower incidence and the predominant framing of HPV as a women’s health issue—represents a further preventable HPV-attributable malignancy and reinforces the rationale for gender-neutral vaccination [2,4,5].

From a clinical perspective, the progression from persistent infection with high-risk strains to cervical intraepithelial neoplasia and, subsequently, to invasive carcinoma is a predictable process. This evolution offers an extended window of opportunity for intervention, requiring a dual strategy: primary vaccination for protecting against the etiological agent and screening for the early detection of lesions [3,4,6]. Nevertheless, the universal validity of this biological model is frequently eclipsed by public health failures [3,5].

Cervical cancer mortality rates serve as the ultimate indicator of the efficacy of a national health system, measuring the capacity of medical infrastructure to translate scientific innovation into clinical protocols. In Romania, the epidemiological profile remains critical, characterised by an alarming incidence of newly diagnosed cases, frequently in advanced stages, which inevitably leads to high mortality rates [1,4]. This reality contrasts sharply with Western European nations such as the United Kingdom, Portugal, and Norway, where organised, predominantly school-based HPV vaccination programmes have achieved coverage at or above the WHO 90% threshold and produced a marked reduction in the incidence of high-grade cervical lesions and invasive cervical cancer—demonstrating the clinical effectiveness of population-level prophylaxis [6,7]. In England, the national programme has been associated with a near-elimination of cervical cancer in the earliest vaccinated birth cohorts [6], while Portugal and Norway are among the first EU/EEA countries to reach the 90% coverage target in girls by age 15 [7]. These discrepancies emphasise that high mortality in Romania is not a biological fatality, but a direct consequence of structural barriers and inequity in access to prevention [8,9].

Romania’s epidemiological profile is characterised by uncontrolled incidence, exacerbated by risk co-factors such as smoking and obesity [10,11]. According to the 2023 ICO/IARC fact sheet for Romania, the age-standardised incidence rate stands at 22.6 per 100,000 women, with HPV types 16 and 18 detected in over 84.7% of invasive cancer [12]. Nevertheless, the fundamental vulnerability stems from the failure of primary prevention, as human papillomavirus (HPV) vaccination coverage remains critically below the optimal thresholds of 90% established by the World Health Organization [4,13,14,15]. This discrepancy is fuelled by a fragmented legislative framework based on “opt-in” systems, where patients must proactively navigate a complex bureaucratic system [12,13]. By comparison, successful infrastructures in Western Europe utilise “opt-out” school-based vaccination [7,16].

The “opt-in” model transfers the entire responsibility for initiating preventive action to the patient, generating a fragmented public health architecture [16]. In Romania, obtaining the HPV vaccine requires a proactive attitude: the patient must independently seek information, navigate the bureaucratic system to obtain a prescription, and frequently manage logistical lapses in pharmacies [17,18]. This approach transforms prevention into an obstacle course. Superimposed on variable levels of health literacy and massive exposure to online misinformation, the “opt-in” system directly penalises vulnerable populations [19,20,21]. Essentially, this model does not protect decisional autonomy but rather amplifies social inequities, limiting access to prophylaxis only to segments with high education and resources—a phenomenon that directly explains the marginal national vaccination rate of approximately 6% reported by the WHO for girls aged 15 years in 2023 [20,22,23].

In opposition, the “opt-out” model represents the gold standard in European countries with superior clinical outcomes, grounded in the systemic integration of prevention [8,16]. In this public policy scenario, HPV vaccination is set as the default norm, usually conducted through school immunisation programs [16]. The administrative burden is fully assumed by the state: the medical act is automatically scheduled, and—under the logic of a default-based model—its cancellation occurs only if the parent or patient records an explicit refusal, the precise legal form of which varies between jurisdictions [16]. By eliminating logistical barriers (appointments, prescriptions, travel) and normalising vaccination within the educational environment, “opt-out” systems effectively counteract behavioural inertia [16,24].

Beyond the programmes already discussed (England, Portugal and Norway [6,7]), Belgium is illustrative: with the same vaccine available nationwide, coverage reached ~91% in Flanders, which uses a school-based (opt-out) programme, versus ~36% in the French-speaking Community without equivalent school-based delivery—confirming that the delivery model, not mere availability, often drives compliance [25]. Relying on high adherence from vulnerable cohorts without addressing systemic barriers to access and misinformation constitutes an ineffective approach to preventive care [15,26].

Transitioning toward an efficient cancer control system requires a profound understanding of the multidimensional determinants of health-seeking behaviour [6]. Inequity in access to prophylaxis cannot be explained exclusively by deficient infrastructure; it must be analysed at the intersection of health literacy, risk perception, and the influence of the digital ecosystem [27]. Recent literature suggests that vaccine hesitancy and lack of screening adherence are complex phenomena, often fuelled by major conceptual confusions—such as the inability to distinguish between the utility of cytological diagnosis (Papanicolaou test) and specific HPV testing [28,29]. Furthermore, in the digital era, medical decision-making is profoundly altered by the phenomenon of infodemic exposure [20]. Although prior literature suggests that exposure to anti-vaccination content erodes institutional trust, the magnitude and direct behavioural impact of such exposure remain contested [21]. The present study tests whether this association is detectable at the level of vaccination intention and safety perception.

In this context, the purpose of the present study is to investigate the cognitive and behavioural determinants influencing cervical cancer prevention among the population. We hypothesise that a higher level of accurate HPV-related knowledge is directly associated with a greater intention to receive vaccination, whereas lower educational attainment is linked to both reduced knowledge and higher susceptibility to misinformation. Furthermore, adherence to cervical cancer screening is expected to correlate with superior HPV-related literacy and a higher degree of trust in official health information sources.

## 2. Materials and Methods

This study aimed to examine the associations between HPV-related knowledge, institutional trust, perceived HPV vaccine safety, social media exposure, vaccination attitudes, and preventive healthcare behaviours in women attending outpatient obstetrics and gynaecology clinics. The study also investigated whether higher HPV-related knowledge and trust in official health information sources were associated with greater HPV vaccination uptake and cervical cancer screening adherence.

### 2.1. Participants and Procedure

The present research was designed as an observational, cross-sectional, descriptive study conducted between 1 December 2025 and 31 March 2026 in four outpatient obstetrics and gynaecology ambulatory clinics. Participants were recruited from four outpatient obstetrics and gynaecology clinics in north-western Romania, comprising both public (state-funded) and private units. Three centres were located in Cluj-Napoca (Cluj County), a major urban university city, and one in Beclean (Bistrița-Năsăud County), a small town serving a predominantly rural catchment area. This combination of public and private settings across an urban university centre and a smaller town with a surrounding rural population was intended to capture a broader socioeconomic range of attendees.

Eligibility criteria (women aged 18–65 years) were displayed before questionnaire access. Data were collected anonymously via a self-administered QR-code questionnaire, and only complete, submitted responses were recorded by the platform; partially completed or abandoned questionnaires were not stored, and no data were captured for non-participants. The age range of the cohort reflects the natural case-mix of the participating adult outpatient obstetrics and gynaecology clinics: patients under 18 years are generally referred to paediatric services and rarely attend these consultations, whereas women over 65 years typically fall outside the routine cervical cancer screening window. A convenience sampling approach was employed, with consecutive recruitment across all participating clinics throughout the study period. The combination of public and private units across an urban university centre and a smaller town serving a rural catchment area was intended to broaden the range of clinical and socioeconomic backgrounds represented among routine gynaecological attendees; it was not intended, and by virtue of its non-probability design cannot be assumed, to achieve statistical representativeness of the general female population.

Data collection was anonymous, and no personally identifiable information, including names, email addresses, or IP addresses, was collected. Participation was voluntary, and respondents could discontinue questionnaire completion at any time without affecting medical care.

All procedures were conducted in accordance with the ethical standards of the institutional research committee and the Declaration of Helsinki. Ethical approval was obtained from the local Ethics Committee (IH76442 from 9 August 2025) prior to participant recruitment. Electronic informed consent was obtained from all participants before questionnaire initiation through mandatory confirmation of a consent statement displayed on the first page of the survey.

### 2.2. Data Collection Method

Data collection was conducted via a digitally administered structured questionnaire hosted on a secure web-based platform. Eligible patients were recruited during routine registration in outpatient obstetrics and gynaecology clinics, where they were provided with a unique Quick Response (QR) code to access the survey via personal mobile devices. This digital approach was implemented to ensure real-time data validation, maintain participant anonymity, and optimise response rates within the clinical setting.

The questionnaire was based on previously validated HPV-related assessment instruments, including components derived from the HPV Knowledge Scale, HPV General Knowledge Scale, and HPV Vaccination Knowledge instruments. The survey evaluated HPV-related knowledge, trust in official health information sources, perceived HPV vaccine safety, vaccination intention, exposure to negative HPV vaccine information on social media, preventive healthcare behaviours, and demographic characteristics.

The questionnaire included dichotomous true/false items, Likert-type attitudinal scales, ordinal frequency measures, and binary behavioural variables. The complete instrument, including all items, response formats and scoring rules, is provided in Supplementary Material S1 (original version).

### 2.3. Variables

The questionnaire included four categories of variables: HPV-related knowledge variables, attitudinal variables, preventive behaviour variables, and demographic characteristics. Multi-item constructs were operationalised using composite scores, while attitudinal dimensions were assessed using Likert-type scales. Higher values generally reflected greater levels of the underlying construct, including HPV-related knowledge, institutional trust, perceived vaccine safety, and favourable vaccination attitudes. All these variables are described in Table 1.

HPV-related knowledge was conceptualised as a multidimensional construct reflecting knowledge regarding HPV transmission, HPV-associated cancers, HPV testing, vaccination, and common misconceptions. This construct was operationalised through the composite HPV knowledge score (HPV_KNOW), which was calculated as the sum of 36 true/false items. Each item was coded as 1 for correct responses and 0 for incorrect or “don’t know” responses. The included items assessed medically relevant information such as HPV transmission through sexual contact, the association between HPV infection and cervical cancer, HPV-related oral and anal cancers, vaccine effectiveness, and HPV screening procedures. Higher HPV_KNOW values indicated greater HPV-related knowledge. Participants were additionally categorised into low, moderate and high HPV-related knowledge groups (KNOW_CAT) according to predefined thresholds applied to the standardised knowledge percentage score (KNOW_PCT): low (<50%), moderate (50–74%) and high (≥75%).

Perceived HPV vaccine safety (SAFE_SC) was operationalised as an attitudinal construct reflecting participants’ subjective perceptions regarding the safety and reliability of HPV vaccination. The variable was measured using a five-point Likert scale ranging from 1 (“not safe at all”) to 5 (“very safe”). This construct reflects concerns related to vaccine adverse effects, confidence in vaccine development, and general perceptions of vaccine-related risk.

Trust in official health information sources (TRUST_SC) was included as a core psychological and informational construct reflecting confidence in physicians, public health institutions, the World Health Organization, and governmental health communication. The variable was measured using a five-point Likert scale ranging from 1 (“no trust at all”) to 5 (“very high trust”). This construct was theoretically included due to its established importance in shaping vaccination attitudes and preventive health behaviours.

HPV vaccination intention (INTENTSC) was conceptualised as the participant’s self-reported willingness to receive HPV vaccination. The construct was measured using a five-point Likert scale ranging from 1 (“definitely would not vaccinate”) to 5 (“definitely would vaccinate”). Higher scores indicated stronger behavioural intention toward HPV vaccination.

Exposure to negative HPV vaccine information on social media (NEG_INFO) was operationalised as an ordinal variable reflecting the frequency with which participants encountered negative or potentially misleading HPV vaccine-related content online. The variable was measured using a five-point scale ranging from minimal exposure to very frequent exposure. This construct was included due to the increasing role of social media in shaping vaccination attitudes and public health perceptions.

The composite pro-vaccination attitude score (VAX_ATT) was calculated as the arithmetic mean of perceived vaccine safety (SAFE_SC), trust in official information sources (TRUST_SC), and HPV vaccination intention (INTENTSC). This composite construct was intended to capture the overall attitudinal orientation toward HPV vaccination and preventive healthcare engagement.

Preventive behaviour variables included HPV vaccination status (HPV_VACC) and cervical cancer screening adherence (PAP_TEST). Both variables were operationalised as binary outcomes coded as 1 for affirmative responses and 0 otherwise. These variables were included as indicators of actual preventive healthcare behaviours rather than attitudinal predispositions.

Demographic and behavioural variables were included as control variables in the analyses to account for potential confounding effects. These variables comprised age (AGE_YRS), urban residence (URBAN), relationship status (IN_REL), parental status (HAS_CHILD), smoking behaviour (SMOKER), alcohol consumption (ALCOHOL), age at sexual debut (SEX_DEB), and educational attainment (HIGHER_EDU). Educational attainment was operationalised as a binary variable distinguishing participants with university or postgraduate education from those with lower educational levels. These variables were included because of their potential influence on HPV-related knowledge, preventive attitudes, and healthcare behaviours.

### 2.4. Statistical Analysis

Statistical analyses were performed using non-parametric and correlational approaches considered appropriate for the distribution and structure of the dataset. Continuous variables, including AGE_YRS, HPV_KNOW, SAFE_SC, TRUST_SC, INTENTSC, and VAX_ATT, were summarised using minimum values, maximum values, means, and standard deviations. Categorical and binary variables, including URBAN, HPV_VACC, PAP_TEST, HIGHER_EDU, HAS_CHILD, SMOKER, and ALCOHOL, were expressed as frequencies and percentages.

The composite HPV-related knowledge score (HPV_KNOW) was calculated as the sum of all correctly answered HPV-related true/false items:

(1) $$\mathrm{HPV\_KNOW}=\sum_{i=1}^{36}K_{i}$$

where $K_{i}=1$ for correct responses and $K_{i}=0$ for incorrect or “don’t know” responses.

Participants were subsequently categorised into low, moderate, and high HPV-related knowledge groups (KNOW_CAT) according to predefined thresholds based on the total HPV-related knowledge score.

The composite pro-vaccination attitude score (*VAX*_*ATT*) was calculated as the arithmetic mean of perceived vaccine safety, trust in official information sources, and HPV vaccination intention:

(2) $$\mathrm{VAX\_ATT}=\frac{\mathrm{SAFE\_SC}+\mathrm{TRUST\_SC}+\mathrm{INTENTSC}}{3}$$

Internal consistency of the composite HPV-related knowledge scale was evaluated using Cronbach’s alpha coefficient:

(3) $$\alpha =\frac{k}{k-1}(1|\frac{\sum \sigma_{i}^{2}}{\sigma_{T}^{2}})$$

where k represents the number of items included in the scale, $\sigma_{i}^{2}$ represents item variance, and $\sigma_{T}^{2}$ represents total score variance.

Because all HPV knowledge items were dichotomously scored (correct = 1, incorrect/“don’t know” = 0), Cronbach’s alpha is equivalent to the Kuder–Richardson Formula 20 (KR-20). Internal consistency was evaluated using Cronbach’s alpha, accompanied by a 95% confidence interval (bootstrap estimation). Corrected item–total correlations and alpha-if-item-deleted statistics were additionally examined to assess the contribution of individual items to overall scale reliability.

Because several variables demonstrated non-normal distributions and ordinal scaling, Spearman rank-order correlation coefficients (ρ) were used to evaluate associations between HPV-related knowledge, trust in official information sources, perceived vaccine safety, vaccination intention, and exposure to negative HPV vaccine information on social media. Correlation strength was interpreted according to conventional thresholds for weak, moderate, and strong associations.

Comparisons between vaccinated and non-vaccinated participants were performed using the Mann–Whitney U test. Group comparisons according to HPV-related knowledge categories were conducted using the Kruskal–Wallis test for continuous and ordinal variables and the Chi-square test for categorical variables. The Mann–Whitney U statistic was calculated according to:

(4) $$U=n_{1}n_{2}+\frac{n_{1}(n_{1}+1)}{2}-R_{1}$$

where $n_{1}$ and $n_{2}$ represent sample sizes for the compared groups and $R_{1}$ represents the rank sum for the first group.

Spearman’s correlation coefficient was calculated using the following equation:

(5) $$\rho =1-\frac{6\sum d_{i}^{2}}{n(n^{2}-1)}$$

where $d_{i}$ represents the difference between paired ranks and n represents the number of paired observations. All statistical tests were two-tailed, and statistical significance was established at p<0.05.

No a priori sample-size calculation was performed, as the study used consecutive convenience recruitment over a fixed period. A post hoc consideration indicates that the achieved sample (N = 441) provides adequate power for the principal Spearman correlation and Mann–Whitney/Kruskal–Wallis analyses, but limited power for inference within small subgroups (e.g., low-knowledge or under-served strata); subgroup findings are therefore reported descriptively and interpreted with caution.

## 3. Results

### 3.1. Descriptive Statistics

During the study period, between December 2025 and March 2026, a total of 441 eligible female patients successfully completed the digital questionnaire across the four participating outpatient obstetrics and gynaecology clinics. No questionnaire accesses outside the 18–65-year range were registered during the study period; consequently, the 441 complete questionnaires represent both the number of participants assessed and the final number included, with no recordable post-access exclusion stage. The study population had a mean age of 32.20 years (SD = 8.81), with ages ranging from 19 to 64 years. Most participants lived in urban areas (URBAN = 1 in 78.2% of cases), while 72.3% reported being married or currently in a relationship (IN_REL = 1). Approximately half of the respondents had children (HAS_CHILD = 48.8%). Current smoking was reported by 27.0% of participants (SMOKER = 1), whereas alcohol consumption (ALCOHOL = 1) was reported by 24.9% of the sample. HPV vaccination uptake was relatively low, with only 28.6% of respondents reporting prior HPV vaccination (HPV_VACC = 1). Perceived HPV vaccine safety (SAFE_SC) showed relatively high values, with a mean score of 4.32 (SD = 0.98) on a 1–5 Likert scale. Similarly, trust in official health information sources (TRUST_SC) was high, with a mean of 4.24 (SD = 1.06). HPV vaccination intention (INTENTSC) also demonstrated elevated values, with a mean score of 4.03 (SD = 1.28). Exposure to negative HPV vaccine information on social media (NEG_INFO) was frequent, as 51.9% of participants reported high or very high exposure levels (categories 4–5). Regarding sexual behaviour, 57.1% of respondents reported a sexual debut after the age of 18 years (SEX_DEB = 3), whereas only 10.9% reported sexual debut before the age of 16 years (SEX_DEB = 1). The composite HPV knowledge score (HPV_KNOW) ranged from 0 to 36, with a mean of 22.64 (SD = 9.17), indicating moderate overall HPV-related knowledge within the study population. The standardised knowledge percentage score (KNOW_PCT) had a mean value of 62.89% (SD = 25.47). Based on KNOW_CAT, 38.5% of participants demonstrated high HPV-related knowledge, while 26.8% were classified as having low knowledge levels. The composite pro-vaccination attitude score (VAX_ATT) demonstrated generally favourable attitudes toward HPV vaccination, with a mean value of 4.20 (SD = 0.96). Higher educational attainment (HIGHER_EDU = 1) was identified in 27.4% of respondents.

### 3.2. Graphical Analysis of Main Study Variables

**Figure 1:**
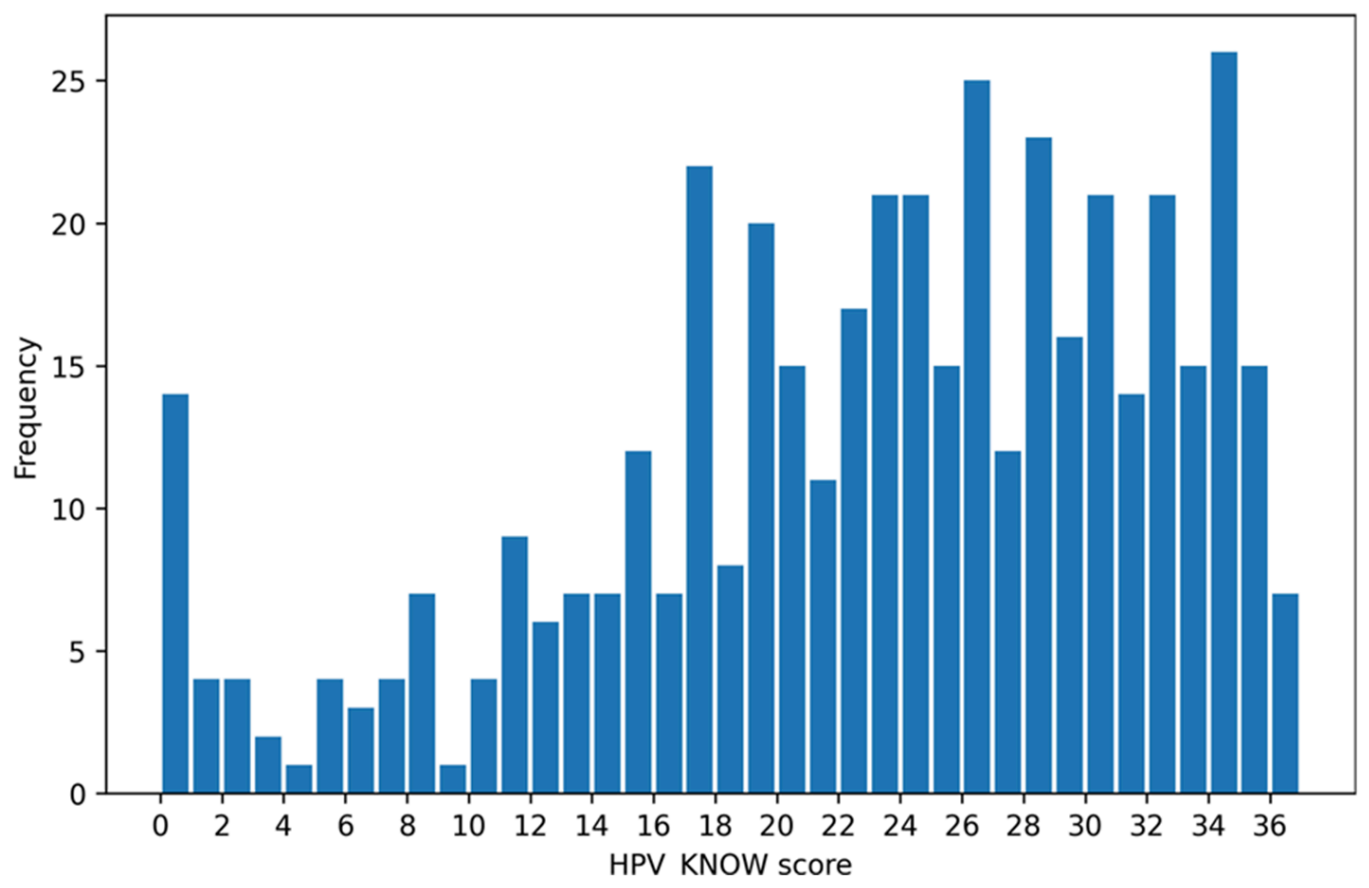
Distribution of the Composite HPV Knowledge Score (HPV_KNOW) in the study population. Note: Bars represent the number of participants (frequency) for each HPV_KNOW score. Higher scores indicate greater HPV-related knowledge, while the distribution illustrates the variability of knowledge levels across the sample.

Figure 1 illustrates the distribution of the composite HPV knowledge score (HPV_KNOW) within the study population. The distribution was moderately skewed toward higher values, with most participants scoring between 20 and 35 points. Higher HPV_KNOW values indicate greater HPV-related knowledge, while the overall shape of the distribution reflects the variability of health literacy levels across the sample. The mean HPV_KNOW score was 22.64 (SD = 9.17), suggesting an overall moderate level of HPV-related knowledge. Nevertheless, the presence of very low scores, including several participants with values close to 0, indicates substantial heterogeneity in HPV-related literacy across the sample. The wide spread of scores demonstrates that participants differed considerably in their understanding of HPV-related topics, supporting the discriminatory capacity of the scale. The relatively broad distribution supports the use of HPV_KNOW as a continuous variable in subsequent analyses examining associations between knowledge, trust, vaccination intention, and preventive behaviours.

**Figure 2:**
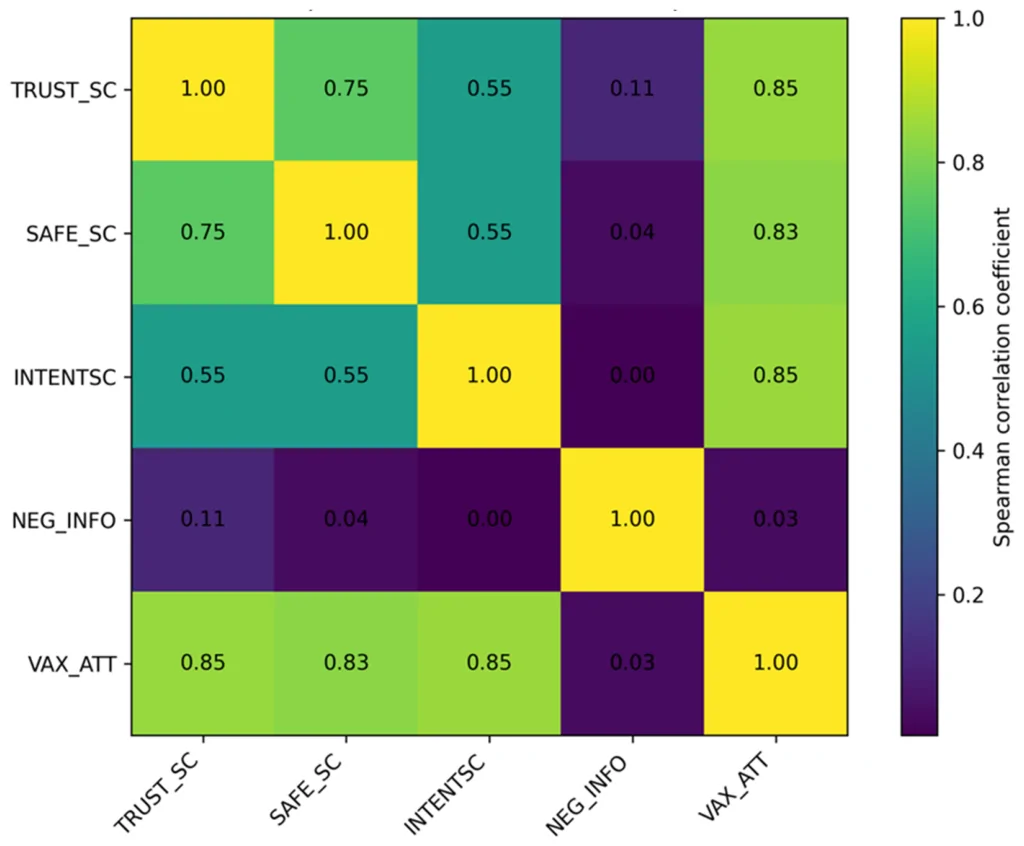
Spearman correlation heatmap of HPV-related knowledge, attitudes, and information exposure variables. Note: Each cell displays the Spearman correlation coefficient (ρ) between two variables. Darker colour intensity indicates stronger positive correlations, whereas lighter colours indicate weaker associations.

Figure 2 presents the Spearman correlation heatmap for the principal attitudinal and knowledge-related variables. Darker colour intensities indicate stronger positive correlations, whereas lighter colours indicate weaker associations between variables. Strong positive correlations were observed between TRUST_SC and SAFE_SC (ρ = 0.75), as well as between TRUST_SC and VAX_ATT (ρ = 0.85). Similarly, SAFE_SC was positively associated with INTENTSC (ρ = 0.55), while INTENTSC demonstrated a strong positive correlation with the composite pro-vaccination attitude score (VAX_ATT; ρ = 0.85). In contrast, exposure to negative vaccine-related information on social media (NEG_INFO) showed only weak correlations with the remaining variables (ρ ranging between 0.00 and 0.11). The concentration of stronger correlations among TRUST_SC, SAFE_SC, INTENTSC, and VAX_ATT indicates that these constructs are closely interconnected and may jointly influence vaccination attitudes. Overall, the heatmap suggests that trust in official information sources and perceived vaccine safety are strongly related to vaccination intention and favourable vaccination attitudes.

**Figure 3:**
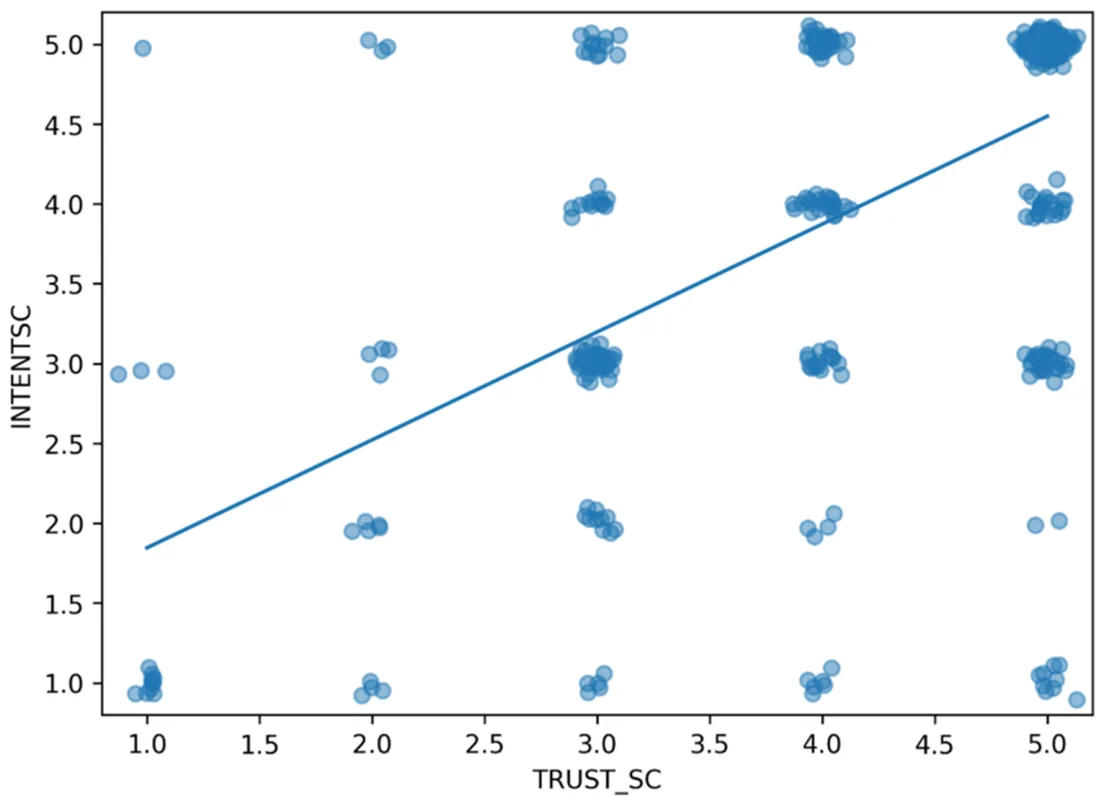
Association between trust in official health information sources (TRUST_SC) and HPV vaccination intention (INTENTSC). Note: Each point represents one participant. The fitted trend line illustrates the positive association between institutional trust and vaccination intention, with higher TRUST_SC values generally corresponding to higher INTENTSC scores.

The positive association between trust in official health information sources (TRUST_SC) and HPV vaccination intention (INTENTSC) is demonstrated in Figure 3. Each point represents an individual participant, while the upward trend indicates that higher levels of institutional trust are associated with greater willingness to receive HPV vaccination. Participants with TRUST_SC values of 4–5 predominantly displayed INTENTSC scores between 4 and 5, whereas lower trust scores were more frequently associated with low or moderate vaccination intention. The clustering of observations in the upper-right portion of the graph further highlights the coexistence of high trust and strong vaccination intention among many respondents. This pattern suggests that confidence in physicians, public health authorities, and official health communication may play a major role in shaping preventive vaccination attitudes.

**Figure 4:**
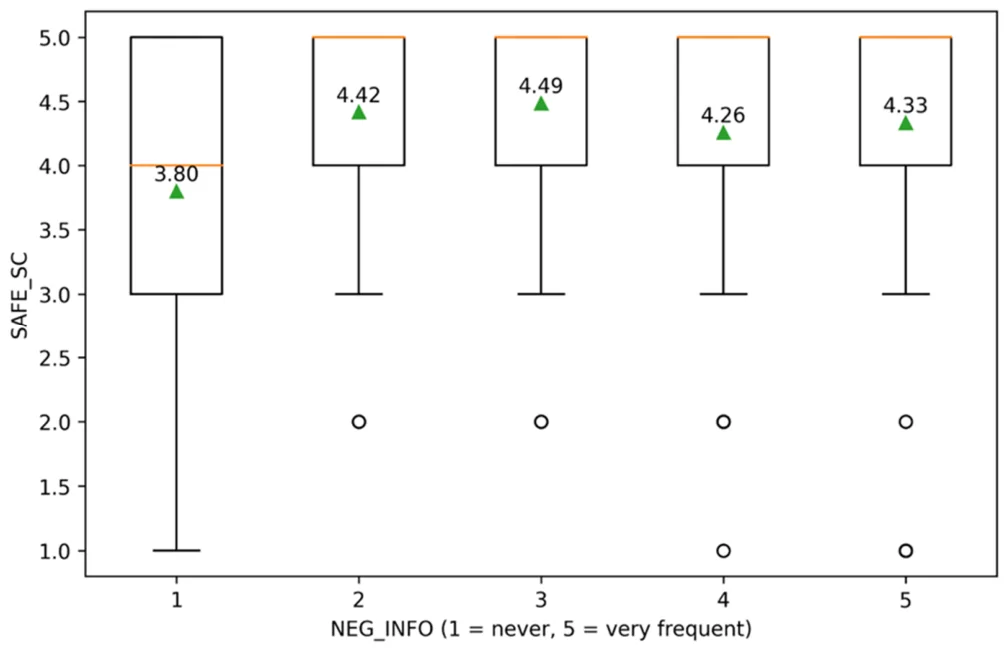
Perceived HPV vaccine safety (SAFE_SC) according to exposure to negative HPV vaccine information on social media (NEG_INFO). Note: The box represents the interquartile range (IQR), the horizontal line within the box indicates the median, and the whiskers represent the overall spread of the data excluding outliers. Individual circles denote outliers, and the green triangle indicates the mean value for each exposure category.

Figure 4 depicts the distribution of perceived HPV vaccine safety (SAFE_SC) according to exposure to negative HPV vaccine information on social media (NEG_INFO). In the boxplot, the central line represents the median, the box indicates the interquartile range, and the whiskers illustrate the overall spread of responses within each exposure category. Participants reporting minimal exposure to negative online information (NEG_INFO = 1) had a lower mean SAFE_SC value (mean = 3.80), whereas individuals in categories 2–5 demonstrated mean safety perception scores ranging from 4.26 to 4.49. Despite the absence of a strictly linear decline across categories, the figure highlights variability in vaccine safety perception according to social media exposure patterns. The relatively narrow boxes concentrated around values of 4–5 indicate that most participants reported favourable perceptions of HPV vaccine safety regardless of their level of exposure to negative online content. The boxplot additionally demonstrates a relatively compressed distribution of SAFE_SC values around high scores, suggesting that most respondents maintained generally positive perceptions regarding HPV vaccine safety despite varying exposure to online negative information.

### 3.3. Reliability Analysis

The HPV knowledge scale (HPV_KNOW), composed of 36 dichotomous true/false items, demonstrated excellent internal consistency. Cronbach’s alpha was 0.939 (95% CI: 0.929–0.948), indicating a high degree of homogeneity among the items. Because all items were dichotomously scored, this value is equivalent to the Kuder–Richardson Formula 20 (KR-20).

Corrected item–total correlations ranged from 0.245 to 0.663, demonstrating that all items contributed positively to the overall construct. The lowest corrected item–total correlation was observed for item HPVK09 (r = 0.245), whereas the highest was observed for item HPVK02 (r = 0.663).

The alpha-if-item-deleted analysis showed values ranging between 0.936 and 0.940, indicating that removal of any individual item would not meaningfully improve scale reliability. These findings support the retention of all 36 knowledge items and confirm the reliability of the HPV knowledge construct for subsequent correlational and group-comparison analyses.

The internal consistency analysis of the HPV knowledge scale demonstrated excellent reliability. Detailed reliability indicators, including Cronbach’s alpha, confidence intervals, and item–total correlation statistics, are presented in Table 2.

### 3.4. Spearman Correlation Analysis

Spearman correlation analysis was performed to explore the associations between HPV-related knowledge, trust in official information sources, perceived vaccine safety, vaccination intention, and exposure to negative HPV vaccine information on social media. The analysis revealed several statistically significant positive correlations between the main attitudinal and knowledge-related variables.

A moderate positive correlation was identified between the composite HPV knowledge score (HPV_KNOW) and trust in official health information sources (TRUST_SC) (ρ = 0.35, *p* < 0.001), suggesting that participants with higher HPV-related knowledge tended to report greater confidence in physicians, public health authorities, and institutional health communication. Similarly, HPV_KNOW demonstrated significant positive correlations with perceived HPV vaccine safety (SAFE_SC) (ρ = 0.31, *p* < 0.001) and HPV vaccination intention (INTENTSC) (ρ = 0.33, *p* < 0.001). These findings indicate that higher levels of accurate HPV-related knowledge were associated with more favourable attitudes toward HPV vaccination and greater willingness to receive the vaccine.

The strongest associations were observed among the attitudinal variables. TRUST_SC showed a strong positive correlation with SAFE_SC (ρ = 0.75, *p* < 0.001), indicating that greater trust in official information sources was closely associated with higher perceived vaccine safety. In addition, TRUST_SC was moderately correlated with INTENTSC (ρ = 0.55, *p* < 0.001), while SAFE_SC also demonstrated a moderate positive association with INTENTSC (ρ = 0.55, *p* < 0.001). These findings suggest that institutional trust and vaccine safety perceptions may represent key psychological determinants of HPV vaccination intention.

As expected, the composite pro-vaccination attitude score (VAX_ATT) demonstrated very strong positive correlations with the variables used in its construction, namely INTENTSC (ρ = 0.85, *p* < 0.001), TRUST_SC (ρ = 0.85, *p* < 0.001), and SAFE_SC (ρ = 0.83, *p* < 0.001). These associations primarily reflect the internal coherence of the composite measure and should not be interpreted as independent relationships between separate constructs.

Exposure to negative HPV vaccine information on social media (NEG_INFO) demonstrated comparatively weaker associations with the remaining variables. A weak negative correlation was observed between NEG_INFO and HPV_KNOW (ρ = −0.10, *p* = 0.032), suggesting that participants more frequently exposed to negative vaccine-related content tended to have slightly lower HPV-related knowledge levels. However, NEG_INFO did not demonstrate statistically significant associations with either SAFE_SC (ρ = 0.04, *p* = 0.457) or INTENTSC (ρ = 0.005, *p* = 0.923), indicating that exposure to negative information alone may not directly influence vaccine safety perception or vaccination intention within the present sample.

The correlation analysis suggests that HPV-related knowledge, institutional trust, and perceived vaccine safety are closely interconnected constructs and are positively associated with favourable HPV vaccination attitudes and behavioural intentions.

### 3.5. Group Comparisons According to HPV Vaccination Status

Group comparison analyses revealed significant differences between vaccinated and non-vaccinated participants across several HPV-related knowledge and attitudinal variables. Participants reporting prior HPV vaccination (HPV_VACC = 1) demonstrated higher HPV_KNOW scores compared to non-vaccinated individuals (27.53 ± 7.63 vs. 20.68 ± 9.02, *p* < 0.001), suggesting that accurate HPV-related knowledge may be associated with preventive vaccination behaviour. Vaccinated participants also reported significantly higher levels of trust in official health information sources (TRUST_SC: 4.73 ± 0.59 vs. 4.04 ± 1.15, *p* < 0.001) and more favourable perceptions regarding HPV vaccine safety (SAFE_SC: 4.71 ± 0.58 vs. 4.16 ± 1.06, *p* < 0.001).

Similarly, HPV vaccination intention (INTENTSC) was substantially higher among vaccinated individuals (4.94 ± 0.33) compared to non-vaccinated participants (3.67 ± 1.34, *p* < 0.001), indicating coherence between actual vaccination behaviour and future preventive attitudes. Exposure to negative HPV vaccine information on social media (NEG_INFO) showed smaller between-group differences (3.59 ± 1.34 vs. 3.25 ± 1.17, *p* = 0.004), indicating that online exposure alone may not fully explain vaccination status in the present sample. Overall, these findings support the existence of meaningful associations between HPV-related knowledge, institutional trust, vaccine safety perception, and preventive vaccination behaviour. These data are presented also in Table 3.

### 3.6. Knowledge Category Comparisons

Participants classified in the high HPV knowledge category (KNOW_CAT = 3) demonstrated consistently more favourable attitudinal and behavioural outcomes compared to individuals with low HPV-related knowledge. Mean vaccination intention (INTENTSC) increased progressively across knowledge categories, from 3.46 in the low-knowledge group to 4.48 in the high-knowledge group (Kruskal–Wallis statistic = 48.63, *p* < 0.001). Similarly, perceived HPV vaccine safety (SAFE_SC) was substantially higher among participants with high HPV-related knowledge (4.65) compared to those with low knowledge (3.76, *p* < 0.001).

Trust in official health information sources (TRUST_SC) also demonstrated a significant positive gradient according to HPV knowledge level, increasing from 3.67 in the low-knowledge category to 4.61 in the high-knowledge category (*p* < 0.001). HPV vaccination rates differed considerably between groups, with vaccination prevalence increasing from 10.2% among participants with low HPV knowledge to 50.0% in the high-knowledge category (χ^2^ = 64.76, *p* < 0.001). Pap smear screening adherence (PAP_TEST) also showed higher frequencies among individuals with greater HPV-related knowledge, suggesting a broader association between health literacy and preventive healthcare behaviours. Overall, these findings indicate that higher HPV-related knowledge levels are consistently associated with greater institutional trust, more favourable vaccine perceptions, higher vaccination intention, and increased engagement in preventive behaviours. Detailed analysis is shown in Table 4.

## 4. Discussion

The present study analysed the relationships between HPV-related knowledge, misinformation, institutional trust, perceived vaccine safety, social media exposure, and preventive behaviours related to HPV vaccination and cervical cancer screening. Overall, the findings suggest that HPV-related knowledge and trust in official health information sources represent central factors associated with favourable vaccination attitudes and preventive healthcare behaviours [2,30,31,32,33].

A descriptive epidemiological analysis covering 2001–2019 confirmed that Romania maintains the highest cervical cancer mortality rates in the European Union, with marked rural-urban disparities reflecting differential access to prevention [32,33,34,35,36].

The vaccination uptake observed in our sample (28.6%) is roughly four to five times the national coverage estimate (~6% in 2023). Rather than indicating improving national coverage, this gap is best understood as a direct consequence of recruiting from women already engaged with gynaecological services, who are predisposed toward preventive behaviours. The findings therefore describe the attitudinal determinants of vaccination within a clinically engaged outpatient population and cannot be extrapolated to the general Romanian female population, particularly its rural and under-served segments.

The findings of this study underscore that accurate HPV-related knowledge serves as a fundamental catalyst for preventive health behaviours. Our analysis demonstrates a significant association between health literacy regarding viral transmission and pathology and the intention to undergo vaccination. Notably, participants categorised within the high-knowledge cohort exhibited a substantially greater willingness to receive the vaccine compared to those with limited knowledge [21,31,32,33].

In alignment with these observations, the data revealed that previously vaccinated individuals possess a significantly more robust factual foundation and lower levels of misinformation than non-vaccinated participants. These findings show that, in our cross-sectional sample, lower HPV-related knowledge co-occurred with lower vaccine uptake. The temporal direction of this association cannot be inferred from the present design; vaccinated individuals may also acquire higher post-decisional knowledge through clinical exposure.

Regarding the influence of the digital ecosystem, while participants frequently reported exposure to negative HPV vaccine content on social media, this exposure did not consistently serve as a direct determinant of vaccine safety perceptions or immunisation intent within the studied sample. However, the observed negative correlation between social media consumption and HPV-related knowledge suggests a more insidious effect. Infodemic exposure likely operates indirectly by fostering a climate of uncertainty and eroding the cognitive foundation necessary for informed decision-making, rather than triggering an immediate or overt refusal.

A primary psychological determinant identified in this research is trust in official health information sources. Confidence in physicians, public health authorities, and international organisations demonstrated a robust positive association with both perceived vaccine safety and vaccination intention. Trust, therefore, acts as a pivotal mediator between medical information and behavioural adherence.

These findings resonate with evidence gathered at the primary care level in Romania. A national cross-sectional survey of 209 family physicians revealed that, while 90% reported offering HPV vaccination, the overall attitudinal profile was only moderately positive, with parental hesitancy identified as the primary barrier by 81.8% of respondents and a majority of providers requesting additional training in vaccine communication and misinformation management. This pattern confirms that the erosion of institutional trust does not originate exclusively from patients but is compounded by gaps in the communication capacity of frontline healthcare providers [18,33,34].

It is also important to note that our sample was recruited from specialised obstetrics and gynaecology consultations rather than from primary care. Women attending such consultations are typically more engaged with reproductive and preventive healthcare, and the clinicians they encounter may hold more favourable, better-informed attitudes toward HPV vaccination than the general family-physician population surveyed nationally. This difference in care setting plausibly contributes to the substantially higher vaccination rate observed here (28.6%) compared with national primary-care-based estimates.

Complementarily, a behavioural study examining the impact of awareness campaigns on cervical cancer screening participation found that, while informational exposure increased understanding of screening utility, logistical and financial barriers remained decisive determinants of actual uptake, with women frequently postponing appointments due to procedural complexity. Taken together, these data suggest that improving vaccination and screening rates requires a dual strategy: strengthening physician-patient trust through structured communication training and simultaneously removing structural access barriers, including simplification of referral pathways and extension of clinic availability [18,33,35,36,37,38,39,40].

The influence of socio-demographic factors appeared more nuanced than initially hypothesised. While lower educational attainment was associated with reduced HPV-related knowledge and less favourable preventive attitudes, the magnitude of this association was limited. Rather than interpreting this as a substantive effect, we note that education was operationalised as a binary indicator (university/postgraduate vs. lower attainment), which constrains its variability and the statistical resolution with which its influence can be assessed. These results suggest that formal education, while protective, does not provide absolute immunity against the complexities of modern vaccine hesitancy [40,41,42].

Also, the study highlights a clear synergy between different modalities of cervical cancer prevention. Participants characterised by higher HPV-related knowledge and greater institutional trust demonstrated not only a higher propensity for vaccination but also superior adherence to screening protocols, such as Papanicolaou testing. These findings suggest that health education and institutional confidence are the foundation that support comprehensive engagement with integrated cervical cancer prevention strategies, facilitating a transition from reactive care to proactive health management [41].

However, the presence of highly informed yet hesitant individuals suggests a “knowledge-intention gap” characterised by decisional inertia. Even when patients possess the cognitive tools to understand the benefits of immunisation, the transition to action is often hindered by emotional barriers or perceived complexity, a phenomenon extensively documented in health psychology as a failure of the “action phase” of decision-making [39,40,41,42].

Romania’s high cervical cancer mortality provides the broader public health context in which our findings should be read. Whether the cognitive deficits observed in our sample contribute causally to national mortality cannot be established from a cross-sectional convenience sample and requires longitudinal evidence. Without a fundamental shift in how HPV risks are communicated and how prevention services are delivered, the clinical infrastructure will remain unable to translate scientific innovation into improved survival rates [11,12,13].

Regarding the role of the primary care physician, our results indicate a concerning shift toward social media as a primary information source. In countries with high vaccination rates, the physician’s recommendation remains the decisive factor. In our sample, the erosion of this influence suggests a need to re-empower medical professionals as the central figures in the immunisation dialogue—a need corroborated by recent national survey data showing that the majority of Romanian family physicians themselves request additional training in vaccine communication, with parental hesitancy named as the dominant barrier to HPV vaccine uptake [17,23,39].

The broader global vaccination coverage landscape further contextualises the urgency of the issues identified in our study. A Joinpoint regression analysis of data reported by 147 countries to WHO and UNICEF documented a significant regression in HPV vaccine coverage during the COVID-19 pandemic, partially reversing gains achieved over the preceding decade, with a strong positive correlation identified between the human development index and vaccination rates. The 2022 WHO position paper on HPV vaccines, developed through systematic evidence review by SAGE, formally endorsed single- and two-dose regimens as cost-effective and logistically feasible strategies for achieving population-level protection and provides the regulatory framework for Romania to implement such catch-up programmes [43,44,45,46].

Finally, evaluating our sample against the WHO 90-70-90 targets reveals a significant distance from the 2030 goals. Our data emphasise that catch-up campaigns for the 18–65 age group are essential, as this demographic remains highly vulnerable to both infection and misinformation, yet is frequently overlooked in primary prevention policies—an observation consistent with global coverage data showing that structural inequity, rather than vaccine refusal alone, is the primary driver of coverage deficits in middle-income countries [47].

### Strengths and Limitations

This study’s primary strength lies in its integration of infodemic exposure and institutional trust as multidimensional variables within the KAP model, providing a more nuanced view of vaccine hesitancy. The use of validated scales (HPV Knowledge Scale) ensures methodological rigour. However, the limitations include a cross-sectional design that prevents causal assertions and a convenience sampling method that may favour participants with higher health engagement. Furthermore, the self-reported nature of the data may introduce social desirability bias. Also, a central limitation is the selection bias arising from convenience sampling within outpatient gynaecology clinics. Women presenting for gynaecological consultation are, by definition, already engaged with preventive healthcare services, which biases the sample toward more health-active behaviours. This is reflected in an HPV vaccination uptake of 28.6%—substantially above the national estimate of approximately 6% for 2023—and in correspondingly high screening adherence.

A further limitation concerns the assessment of educational attainment. Education was captured only as a binary variable (university/postgraduate vs. lower attainment); this coarse operationalisation limits sociodemographic variability and reduces the statistical resolution with which the independent contribution of education can be estimated. Associations involving educational attainment should therefore be interpreted with caution and warrant confirmation in samples with a finer, ordinal characterisation of education.

Consequently, both the absolute prevalence estimates and the strength of the observed associations (particularly for screening adherence) may not generalise to the wider Romanian female population, and least of all to rural or under-served groups. Furthermore, data collection relied exclusively on a QR code accessed via participants’ personal smartphones. This approach, although efficient and privacy-preserving, may have excluded women without a personal smartphone or with limited digital literacy—groups that overlap with the vulnerable populations facing the greatest access barriers—thereby compounding the selection bias and further limiting generalisability.

## 5. Conclusions

This research highlights the complex cognitive factors underlying preventive oncology, identifying health literacy and trust in healthcare institutions as the two primary determinants of vaccine acceptance. The data suggest that while an informed patient is theoretically more inclined toward prophylaxis, the transition from understanding to clinical action is mediated by the perceived legitimacy of health authorities. Consequently, bridging the “intention-behaviour gap” requires more than the mere dissemination of facts; it necessitates a concerted effort to rebuild the patient–provider relationship and streamline the decision-making process within the clinical setting.

Within our sample, exposure to negative HPV vaccine content on social media was not directly associated with vaccination intention or perceived vaccine safety; it correlated only weakly and inversely with factual HPV knowledge. We therefore refrain from concluding that infodemic exposure directly drives vaccine skepticism in this population. The weak inverse association with knowledge does, however, raise the hypothesis that the digital information environment may exert a more diffuse, indirect influence on the factual basis for decision-making—a possibility that warrants dedicated, longitudinal investigation rather than being inferred from the present cross-sectional data.

Furthermore, the evident alignment between screening participation and immunisation status points toward a “holistic prevention” model. By leveraging routine gynaecological encounters as strategic intervention points, healthcare systems can integrate primary and secondary prevention more effectively. Especially in regions with persistent epidemiological disparities, such as Romania, moving away from fragmented, “opt-in” frameworks toward a cohesive, physician-led preventive circuit is imperative for reducing the national burden of cervical cancer.

Because our findings come from adult women (aged 18–65) attending gynaecological consultations, the most directly supported recommendation is to use these routine visits as opportunities for catch-up HPV vaccination and screening, supported by clear, physician-led communication that strengthens trust in official health information. School-based opt-out programmes remain an important national-policy tool for adolescents, as shown in higher-coverage European countries, but they fall outside the population studied here and are mentioned only as policy context, not as a conclusion drawn from our data.

## Figures and Tables

**Table 1:** Sociodemographic characteristics, HPV-related knowledge, attitudes, and preventive behaviours of the study population.

Variable	Variable Explanation	Descriptive Statistics
AGE_YRS	Age in years. Participant age reported in years as a continuous demographic variable.	Min = 19.00; Max = 64.00; Mean = 32.20; SD = 8.81
URBAN	Urban residence. Derived from the question regarding area of residence. Coded as 1 = urban and 0 = rural.	0 = 21.8%; 1 = 78.2%
IN_REL	Married or currently in a relationship. Recoded as 1 if married/in a relationship and 0 otherwise.	0 = 27.7%; 1 = 72.3%
HAS_CHILD	Having children. Derived from the question ‘Do you have children?’. Binary variable coded 1 = yes, 0 = no.	0 = 51.2%; 1 = 48.8%
SMOKER	Current smoking status. Derived from the question ‘Do you currently smoke?’. Binary variable coded 1 = yes, 0 = no.	0 = 73.0%; 1 = 27.0%
ALCOHOL	Alcohol consumption. Derived from the question ‘Do you consume alcohol?’. Binary variable coded 1 = yes, 0 = no.	0 = 75.1%; 1 = 24.9%
HPV_VACC	HPV vaccination status. Derived from the question ‘Have you been vaccinated against HPV?’. Binary variable coded 1 = yes, 0 = no.	0 = 71.4%; 1 = 28.6%
SAFE_SC	Perceived HPV vaccine safety assessed on a 1–5 Likert scale ranging from ‘not safe at all’ to ‘very safe’.	Min = 1.00; Max = 5.00; Mean = 4.32; SD = 0.98
TRUST_SC	Trust in official health information sources (physicians, WHO, Ministry of Health) assessed on a 1–5 Likert scale.	Min = 1.00; Max = 5.00; Mean = 4.24; SD = 1.06
INTENTSC	HPV vaccination intention assessed on a 1–5 Likert scale.	Min = 1.00; Max = 5.00; Mean = 4.03; SD = 1.28
NEG_INFO	Exposure to negative HPV vaccine information on social media. Higher values indicate more frequent exposure.	1 = 9.1%; 2 = 14.7%; 3 = 24.3%; 4 = 21.3%; 5 = 30.6%
SEX_DEB	Age at sexual debut coded as 1 = <16 years, 2 = 16–18 years, 3 = >18 years.	1 = 10.9%; 2 = 32.0%; 3 = 57.1%
HPV_KNOW	Composite HPV knowledge score calculated as the SUM of all HPV true/false knowledge items. Each item coded as 1 = correct and 0 = incorrect/’don’t know’. Higher scores indicate better HPV-related knowledge.	Min = 0.00; Max = 36.00; Mean = 22.64; SD = 9.17
KNOW_PCT	Percentage HPV knowledge score calculated as (correct responses/total HPV knowledge items) × 100.	Min = 0.00; Max = 100.00; Mean = 62.89; SD = 25.47
KNOW_CAT	HPV knowledge category derived from KNOW_PCT: low (<50%), moderate (50–74%), high (≥75%).	1 = 26.8%; 2 = 34.7%; 3 = 38.5%
VAX_ATT	Composite pro-vaccination attitude score calculated as the MEAN of SAFE_SC, TRUST_SC and INTENTSC.	Min = 1.00; Max = 5.00; Mean = 4.20; SD = 0.96
HIGHER_EDU	Higher educational attainment. Binary variable coded as 1 for participants with university/postgraduate education and 0 for lower educational levels.	0 = 72.6%; 1 = 27.4%

Note. Continuous variables are presented as minimum (Min), maximum (Max), mean, and standard deviation (SD). Categorical and binary variables are presented as percentages. HPV_KNOW represents the composite HPV knowledge score calculated as the sum of 36 HPV-related true/false knowledge items (1 = correct answer; 0 = incorrect or “don’t know”). VAX_ATT represents the composite pro-vaccination attitude score calculated as the mean of perceived vaccine safety, trust in official information sources, and vaccination intention scores. Higher scores indicate greater HPV-related knowledge, lower misinformation, and more favourable attitudes toward HPV vaccination.

**Table 2:** Reliability Analysis of the HPV Knowledge Scale (36 items).

Statistic	Value
Number of items	36
Sample size	441
Cronbach’s alpha (KR-20 equivalent)	0.939
95% CI	0.929–0.948
Mean corrected item-total correlation	0.536
Range corrected item-total correlations	0.245–0.663
Range alpha if item deleted	0.936–0.940

Note. HPV_KNOW was composed of 36 dichotomous (true/false) items assessing HPV-related knowledge. Cronbach’s alpha is equivalent to the Kuder–Richardson Formula 20 (KR-20) for dichotomously scored items. Corrected item–total correlations indicate the association between each item and the total scale score excluding that item. Values of Cronbach’s alpha if item deleted represent the estimated reliability coefficient obtained after removing a given item from the scale.

**Table 3:** Comparison of Main Variables According to HPV Vaccination Status.

Variable	HPV_VACC = 0	HPV_VACC = 1	Mann–Whitney U	*p*-Value
HPV_KNOW	20.68 ± 9.02	27.53 ± 7.63	10,318.5	<0.001
TRUST_SC	4.04 ± 1.15	4.73 ± 0.59	13,062.5	<0.001
SAFE_SC	4.16 ± 1.06	4.71 ± 0.58	14,319.5	<0.001
INTENTSC	3.67 ± 1.34	4.94 ± 0.33	8458.5	<0.001
NEG_INFO	3.59 ± 1.34	3.25 ± 1.17	23,228.5	0.004

Note. Values are presented as mean ± standard deviation (SD). *p*-values were calculated using the Mann–Whitney U test.

**Table 4:** Comparisons of Attitudinal and Preventive Variables According to HPV Knowledge Category.

Variable	Low Knowledge	Moderate Knowledge	High Knowledge	Statistic	*p*-Value	Test
INTENTSC	3.46	3.99	4.48	48.63	<0.001	Kruskal–Wallis
SAFE_SC	3.76	4.38	4.65	47.7	<0.001	Kruskal–Wallis
TRUST_SC	3.67	4.26	4.61	53.58	<0.001	Kruskal–Wallis
HPV_VACC	10.2%	19.0%	50.0%	64.76	<0.001	Chi-square

Note. KNOW_CAT was derived from KNOW_PCT and categorised as low (<50%), moderate (50–74%), and high (≥75%) HPV-related knowledge. Continuous variables were compared using the Kruskal–Wallis test, while binary variables were compared using the Chi-square test.

## Data Availability

The data presented in this study are available on request from the corresponding author. The data are not publicly available due to privacy and ethical restrictions regarding patient clinical records.

## Supplementary Materials

The following supporting information can be downloaded at: https://www.mdpi.com/article/10.3390/medsci14040431/s1, Supplementary S1: Questionnaire on the level of knowledge about HPV, HPV testing and HPV vaccination in the general population.

## Abbreviations

The following abbreviations are used in this manuscript:

Abbreviation	Definition
AGE_YRS	Age in years
ALCOHOL	Alcohol consumption (binary variable)
CIN	Cervical Intraepithelial Neoplasia
HAS_CHILD	Parental status (binary variable)
HIGHER_EDU	Higher educational attainment (binary variable)
HPV	Human Papillomavirus
HPV_KNOW	Composite HPV knowledge score
HPV_VACC	HPV vaccination status (binary variable)
IN_REL	Married or currently in a relationship (binary variable)
INTENTSC	HPV vaccination intention score
KAP	Knowledge, Attitudes, and Practices
KNOW_CAT	HPV knowledge category (low/moderate/high)
KNOW_PCT	Percentage HPV knowledge score
NEG_INFO	Exposure to negative HPV vaccine information on social media
PAP_TEST	Cervical cancer (Papanicolaou) screening adherence
QR	Quick Response (code)
SAFE_SC	Perceived HPV vaccine safety score
SD	Standard Deviation
SEX_DEB	Age at sexual debut
SMOKER	Current smoking status (binary variable)
TRUST_SC	Trust in official health information sources score
URBAN	Urban residence (binary variable)
VAX_ATT	Composite pro-vaccination attitude score
WHO	World Health Organization
