# Supplementary material for: The Cervical Cancer Paradox in Eastern Europe: How Knowledge and Institutional Trust Shape HPV Vaccination Attitudes in Romania
Source: Med Sci (Basel). 2026 Jul 25;14(4):431. doi: 10.3390/medsci14040431 (PMC13515567; doi:10.3390/medsci14040431)
Supplement: Supplementary file 1 [file medsci-14-00431-s001.zip › medsci-4374849-supplementary.pdf]

Supplementary S1： Questionnaire on the level of knowledge about HPV, HPV testing and HPV vaccination in the general population

For the following questions, please tick the box corresponding to your answer:

Statement	True	False	Don't know
**Knowledge about HPV**			
HPV is very rare			
HPV always has visible signs or symptoms			
HPV can cause cervical cancer			
HPV can be transmitted through skin-to-skin contact in the genital area			
There are many types of HPV			
HPV can cause HIV/AIDS			
HPV can be transmitted during sexual intercourse			
HPV can cause genital warts			
Men cannot become infected with HPV			
Using a condom reduces the risk of contracting HPV			
HPV can be cured with antibiotics			
Having multiple sexual partners increases the risk of contracting HPV			
Usually, HPV does not require any treatment			
Most sexually active people will have HPV at some point in their life			
A person can have HPV for many years without knowing			
Starting sexual activity at an early age increases the risk of contracting HPV			
HPV can cause anal cancer.			
HPV is a bacterial infection			
HPV can be transmitted through oral sex			
HPV can cause penile cancer			
HPV can cause herpes			
HPV infections always lead to health problems			
HPV can cause oral cancer			
A person without symptoms cannot transmit HPV infection			
**Knowledge about HPV testing**			
An HPV test can show how long you have had the HPV infection			
If a woman has a positive HPV test, she will definitely develop cervical cancer			

The HPV test can be done at the same time as the Pap test			
HPV testing is used to indicate whether the HPV vaccine is needed			
When you take an HPV test, you receive the results the same day			
If an HPV test shows that a woman does not have HPV, her risk of cervical cancer is low			
**Knowledge about HPV vaccination**			
HPV vaccines require three doses			
HPV vaccines provide protection against all sexually transmitted infections			
HPV vaccines are most effective if given to people who have not yet had sexual intercourse			
A person vaccinated against HPV cannot develop cervical cancer			
HPV vaccines provide protection against most cases of cervical cancer			
One of the HPV vaccines provides protection against genital warts			
Girls who have received the HPV vaccine no longer need to have the Pap test as adults			

Have you ever heard about possible side effects of the HPV vaccine?

- ☐ Yes
- ☐ No
- ☐ Not sure

If yes, which of the following side effects have you heard are associated with the HPV vaccine?

(you may tick more than one option)

- ☐ Fever, pain or swelling at the injection site
- ☐ Fatigue, dizziness or general malaise
- ☐ Allergic reactions (e.g. rashes, itching)
- ☐ Neurological problems (e.g. paralysis, loss of consciousness)
- ☐ Infertility / impairment of reproductive function
- ☐ Risk of cancer caused by the vaccine
- ☐ Poisoning with mercury or other heavy metals
- ☐ Other chronic diseases (e.g. autoimmune, thyroid, etc.)
- ☐ I have not heard about side effects
- ☐ Don't know / No answer

What is your perception of the safety of the HPV vaccine on a scale from 1 to 5?

☐1 = not at all safe ☐2 = fairly unsafe ☐3 = medium ☐4 = fairly safe ☐5 = safe

How much trust do you have in official information about the HPV vaccine (from the Ministry of Health, WHO, doctors, etc.)?

☐1 = none ☐2 = low ☐3 = medium ☐4 = high trust ☐5 = very high trust

Intention to vaccinate against HPV on a scale from 1 to 5 (1 = Not at all willing ... 5 = Very willing)

☐1 = I will not get vaccinated ☐2 = I most likely will not get vaccinated ☐3 = I am undecided ☐4 = I probably will get vaccinated☐5 = I will definitely get vaccinated

How often do you come across negative information about the HPV vaccine on social media?

- ☐ Very often
- ☐ Occasionally
- ☐ Rarely
- ☐ Never
- ☐ I do not use social media

To what extent do you consider the information about vaccines on social media to be correct?

- ☐ Most of it is correct
- ☐ Some is correct, some is not
- ☐ Most of it is false or exaggerated
- ☐ I cannot assess

Age at first sexual intercourse :

☐ before 16 years ☐16-18 years ☐after 18 years

Have you ever had a Pap test?

☐ Yes ☐ No ☐ Don't know

If yes, how long ago?

☐ less than 1 year ☐ 1-2 years ☐ over 2 years
